# Effectiveness of a female community health volunteer-led physical activity education intervention on accelerometer-derived outcomes in semi-urban Nepal: an open-label, cluster randomised controlled trial

**DOI:** 10.1186/s12966-026-01894-0

**Published:** 2026-02-23

**Authors:** Rajan Shrestha, Susan Paudel, Anupa Rijal, Tara Ballav Adhikari, Philippe Jean-Luc Gradidge, Bijay Khatri, Dinesh Neupane, Sebastian Dyrup Skejø, Abhinav Vaidya, Taren Sanders, Chris Lonsdale, Narayan Subedi, Sweta Koirala, Per Kallestrup, Rasmus Østergaard Nielsen

**Affiliations:** 1 Department of Public Health, Aarhus University, Aarhus C, Denmark; 2 Nepal Development Society, Kathmandu, Nepal; 3 Department of Paediatrics, Centre for Adolescent Health, University of Melbourne, Parkvile, VIC, Australia; 4 Institute for Physical Activity and Nutrition, School of Exercise and Nutrition Sciences, Deakin University, Geelong, VIC Australia; 5 Department of Sports Science and Clinical Biomechanics, Danish Center for Motivation and Behaviour Science (DRIVEN), University of Southern Denmark, Odense, Denmark; 6 Department of Exercise Science and Sports Medicine, School of Therapeutic Sciences, Faculty of Health Sciences, University of the Witwatersrand, Johannesburg, South Africa; 7 Department of International Health, Bloomberg School of Public Health, Johns Hopkins University, Baltimore, MD USA; 8 Research Unit for General Practice, Aarhus, Denmark; 9 Department of Community Medicine, Kathmandu Medical College, Kathmandu, Nepal; 10 Institute for Positive Psychology and Education, Australian Catholic University, North Sydney, Australia

**Keywords:** Accelerometry, Cluster randomised controlled trial, Female community health volunteers (FCHVs), Nepal, Physical activity promotion

## Abstract

**Background:**

Physical inactivity is a growing public health challenge in low- and middle-income countries (LMICs), yet evidence on scalable, community-based interventions remains limited. We evaluated the effectiveness of a Female Community Health Volunteers (FCHVs)-led, home-based educational programme in promoting physical activity in semi-urban Nepal.

**Methods:**

We conducted a six-month, open-label, cluster-randomised controlled trial among adults in 14 wards of Pokhara, Nepal, with seven clusters each assigned to intervention or control. Trained FCHVs made three home visits, one per month, delivering two-hour sessions promoting physical activity, using materials co-designed with guidance from the Theory of Planned Behaviour. The main outcome was the change in daily device-measured moderate-to-vigorous physical activity (MVPA) from baseline to follow-up.

**Results:**

Among 264 participants (132 in the intervention arm and 132 in the control arm; mean age 49.6 years; 67.5% women) in intention-to-treat analysis, device-measured MVPA declined over six months. However, compared with the control group, the intervention group had a 9.80 min/day smaller decline in non-bout MVPA (95% CI: 0.41–19.18; *p* = 0.041) and a 4.53 min/day smaller decline in MVPA accumulated in ≥ 10-min bouts (95% CI: 0.29–8.77; *p* = 0.036). Positive effects were observed in between-group average acceleration (+ 1.84 mg, *p* = 0.035).

**Conclusions:**

Although MVPA declined in both groups over six months, the FCHV-led, home-based educational intervention attenuated this decline compared to usual care. These findings suggest that existing health volunteer-led education may contribute to maintaining physical activity levels in semi-urban populations.

**Trial registration:**

Trial registration clinicaltrials.gov Identifier: NCT06386692.

**Supplementary Information:**

The online version contains supplementary material available at 10.1186/s12966-026-01894-0.

## Background

Regular physical activity (PA) is essential for health, lowering the risk of cardiovascular disease, type 2 diabetes, various cancers (colon and breast), and premature death, while enhancing cardiorespiratory fitness, mental health, quality of life, and overall well-being [1–3]. Benefits are observed across the life course, and even modest increases in PA can improve health among those who are inactive [4, 5]. Despite these well-established advantages, one in four adults globally does not achieve the World Health Organization (WHO) recommendation of 150–300 min of moderate-intensity or 75–150 min of vigorous-intensity PA weekly [2, 6]. Physical inactivity is a major modifiable risk factor for non-communicable diseases (NCDs), contributing to 0.83 million global deaths [7].

In low- and middle-income countries (LMICs), like Nepal, the prevalence of insufficient PA is increasing, driven by rapid urbanisation, changing work patterns with an increase in sedentary desk work, and reduced active transport [8, 9]. National surveys based on self-reported data suggest that approximately 7% of Nepalese adults in 2019 did not meet the World Health Organization recommendation for PA, compared with about 3% in 2013 [10]. However, these estimates are derived from questionnaire-based assessments, which are known to underestimate the true prevalence of insufficient PA when compared with device-based measures [11, 12]. The burden is higher in semi-urban settings, where more than 40% of adults fail to meet PA recommendations [13]. In these areas, PA is largely work-related, with limited participation in leisure-time PA, a pattern likely to worsen as physically demanding jobs decline [10, 13, 14]. To address this, aligning with the global action plan for PA, Nepal has committed to a 15% relative reduction in insufficient PA by 2030 [15, 16]. This needs national PA guidelines and affordable, culturally-adapted programmes to promote PA, solving common barriers including low awareness, limited motivation, and a lack of supportive environments [17–19]. Community-based educational interventions have demonstrated promising results in managing NCDs in the Nepalese context [20–22]. This approach could also be relevant for PA promotion in similar settings where access to gyms or structured exercise programmes may not be feasible. Contextual PA education delivered by trusted community members can raise awareness, build motivation, and provide practical strategies to integrate PA into daily life.

Female Community Health Volunteers (FCHVs) represent a long-standing, trusted network of grassroots-level health workers in Nepal [23]. Initially focused on maternal and child health, their roles have expanded to include NCD prevention and management [20–23]. Given their reach, cultural familiarity, and integration within primary health systems, they are ideally placed to deliver low-cost, scalable, and contextually relevant PA interventions. Yet, their role in promoting PA has not been tested in rigorously designed randomised controlled trials using device-based PA measures. Therefore, we conducted a cluster randomised controlled trial in semi-urban areas of Pokhara Metropolitan City to assess the effectiveness of an FCHV-led, home-based PA promotion programme compared with a control group. We hypothesised that individuals in the intervention group would observe a greater increase in average MVPA (8.2 min/day) compared to the control group over 6 months.

## Methods

This study adhered to the Consolidated Standards of Reporting Trials (CONSORT) guidelines. It was registered on ClinicalTrial.gov (NCT06386692) and received approval from the Ethical Review Board at the Nepal Health Research Council (726–2023). All participants provided informed written consent. The detailed methods of the trial is described elsewhere [24].

### Study design, setting, and participants

A community-based, open-label, two-group cluster randomised controlled trial was conducted over 6 months in semi-urban areas of Pokhara Metropolitan City (formerly Lekhnath Municipality) located in Gandaki Province, Nepal. The study area comprised all 15 former administrative wards of Lekhnath Municipality, which served as the clusters for randomisation. The trial included baseline and follow-up assessments, with recruitment carried out among participants from the baseline study using the sampling frame of the Community-based management of hypertension in Nepal (COBIN) trial [21].

Of the 15 wards (clusters), one was randomly excluded to achieve balanced allocation between the intervention and control arms. The remaining 14 clusters were randomly allocated to either arm. Participants were then randomly selected from the baseline sampling frame and assigned according to their residential cluster. The participants were invited to participate and were assigned to the respective arm. Clusters were separated geographically to minimise contamination. Some participants received PA education through hypertension and diabetes management trials over eight years ago, which likely had minimal effect due to its limited scope on PA and lack of follow-up [20, 21, 25]. Prior exposure was more common in the intervention group than the control group (64.4% vs 52.3%), although this difference did not reach statistical significance (*p* = 0.061). FCHVs were also directed not to share intervention materials with those outside the intervention group to minimize contamination.

**Fig. 1:**
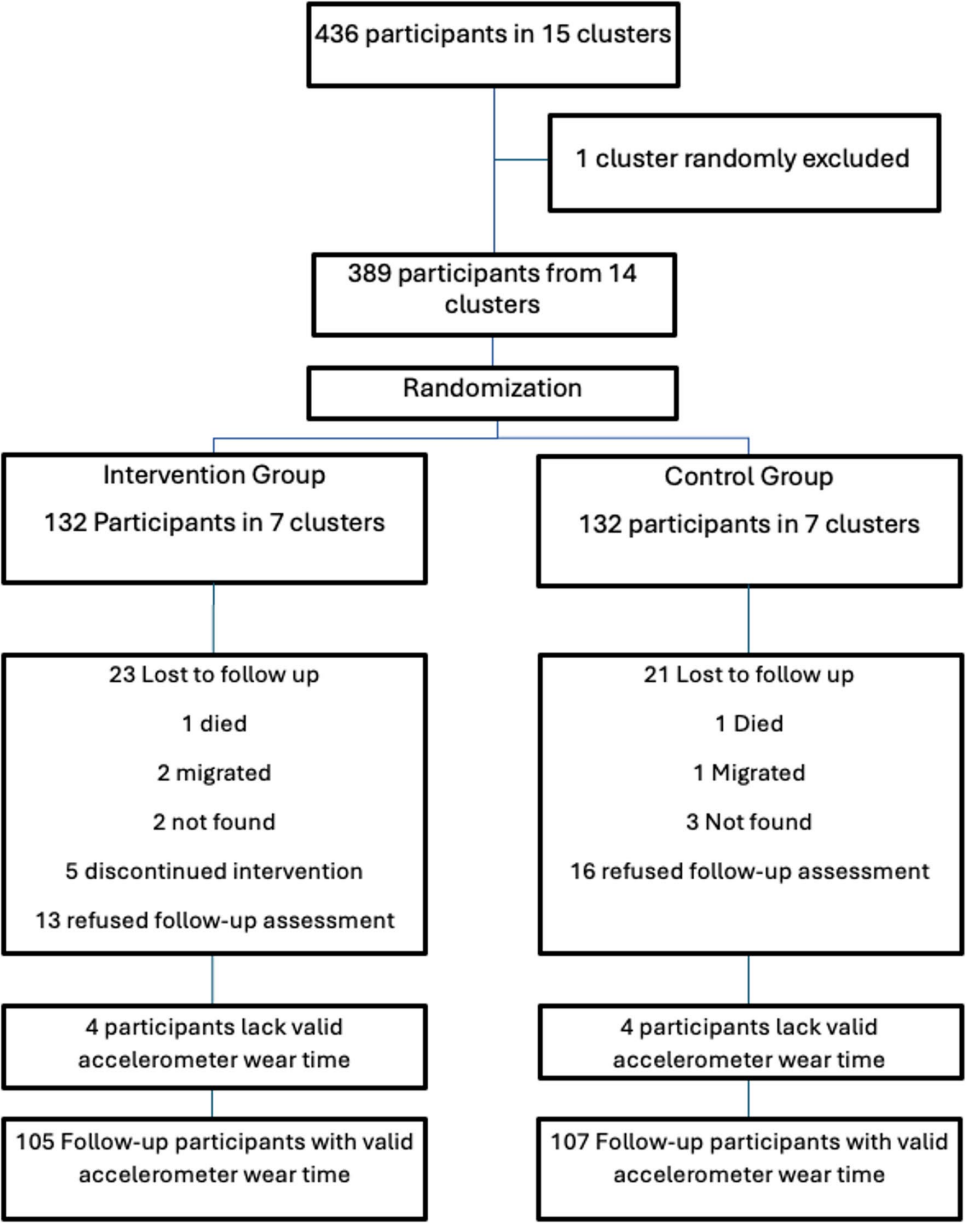
Flow of participants through the trial

Participant selection process is presented in Fig. 1.

### Outcomes

The primary outcome was the change in device-measured MVPA minutes/day from baseline to six-month follow-up. Secondary outcomes included changes in average acceleration measured in Euclidean Norm Minus One (ENMO), light-intensity physical activity (LIPA), sedentary time, PA intention, cardiometabolic indicators, quality of life, and sleep quality.

### Intervention

The intervention consisted of home-based interactive health education sessions through home visits conducted during the first three months by trained FCHVs on community-based PA promotion. Participants in the intervention arm received three face-to-face home visits conducted by trained FCHVs over three months, with one visit per month. These sessions involved discussions on the importance of PA, demonstrations of simple home-based exercises, and distribution and explanation of educational materials such as booklets and manuals. The sessions aimed to educate, motivate, and support families in integrating PA into their daily routines, with an emphasis on simplicity and adherence. Intervention delivery was monitored using participant-level registers that documented the date and time of each home visit and used a checklist to confirm completion of all planned session components. FCHVs were trained through a 3-day interactive training programme, covering non-communicable diseases (NCDs) and their risk factors, the health consequences of insufficient PA, the benefits of regular PA, and strategies for engaging community members. Training methods included interactive workshops, role-playing, and practical sessions to build skills and knowledge.

Before developing the intervention, we conducted focus group discussions with end users to explore their perspectives on PA, including experiences, barriers, and facilitators. The intervention was developed through a user experience-based co-design approach using findings from these discussions and a baseline situation analysis survey. This process included separate workshops with community members and a panel of experts. This panel, comprising public health researchers, physiotherapists, health workers, FCHVs and local citizens, designed a community-based PA promotion package guided by the Theory of Planned Behaviour (TPB), which posits that attitude, subjective norms, and perceived behavioural control influence behavioural intention and ultimately determine behaviour [26–30]. Community members’ input helped to refine intervention content, in terms of language and examples, making them more relevant to the local context and improving feasibility, acceptability, and comprehension. The intervention materials were developed following the P-process, a strategic, evidence-based framework for health communication programme design [31]. A detailed description of intervention components and delivery is provided in Supplementary Table 1, while the intervention development process, training, and implementation feedback are reported in a separate paper [32].

Participation in the intervention was discontinued for individuals who migrated or declined to continue during follow-up. Participants in the control group did not receive any contact, information, education or educational materials from FCHVs until the 6-month follow-up. It was planned to be offered to the wait-list control group. Only the outcome assessor was masked; the data analyst was not masked.

### Data collection and measures

Data were collected by 4 trained research assistants who completed a four-day training programme led by the principal investigator and a public health expert. Household surveys were conducted using a structured questionnaire. Physical measurements included height, weight, waist circumference, and blood pressure, following the protocol used in the STEPS Survey Nepal 2019.

Participants wore a wrist-worn triaxial accelerometer (Axivity AX3, UK) on the non-dominant wrist for seven consecutive days to measure PA, sedentary behaviour, and sleep both during baseline and 6 months follow-up [33, 34]. Devices were set to record at 100 Hz (± 8 g). Data were excluded for participants with fewer than three valid wear days [33]. The ENMO metric was calculated and used to derive Sedentary (< 45 mg), LIPA (45 to 99.9 mg), and MVPA (≥ 100 mg) using established cut points [34–36].

PA intention was measured with four TPB–based items (intention, effort, planning, desire) adapted from prior Nepalese interventions [37]. Responses were rated on a 5-point Likert scale and averaged, with higher scores indicating stronger intentions. We measured cardiometabolic indicators (body weight, height, waist circumference, and blood pressure) using standardized protocols adapted from the WHO STEPS survey [10]. Body mass index (BMI) was calculated as weight (kg) divided by height (m) squared, and waist-to-height ratio (WHtR) was calculated as waist (cm) divided by height (m). Quality of life was assessed using the Nepali version of WHO Quality of Life Brief Version questionnaire, generating domain-specific scores for physical, psychological, social, and environmental well-being [38]. Sleep duration and sleep quality were derived from wrist-worn accelerometer data using GGIR’s default sleep detection algorithm, with sleep quality expressed as the proportion of time spent in good-quality sleep relative to total sleep duration.

### Sample size

Based on the findings from previous studies [39, 40], the sample size was calculated to detect an increase of 8.2 min/day of MVPA in the intervention group compared with the control group, assuming SD = 18, α = 0.05, 80% power, an intra-cluster correlation coefficient of 0.01, and a design effect of 1.39, requiring 106 participants per arm. Allowing for a 20% dropout, 132 participants per arm (264 total) were recruited.

### Data management and statistical analysis

Study data were collected and stored in REDCap with built-in validation rules and daily quality checks. Accelerometers were initialise and data were downloaded using the software called OmGui. Analyses were conducted in R (The R Foundation, 2023) and RStudio (Posit, 2024) under the intention-to-treat principle, with all randomiszed participants analysed in their assigned groups. Multiple imputation by chained equations (MICE) was used for all missing data under the assumption of missing at random, generating 20 imputed datasets with pooled estimates calculated using Rubin’s rules.

The primary analysis estimated the effect of the intervention on change in mean MVPA minutes/day between baseline and six-month follow-up using linear mixed-effects regression models. Model assumptions were assessed with residual normality histograms and Q-Q plots, confirming no violations (Supplementary Fig. 1). Models included fixed effects for time (baseline, follow-up), group (intervention, control), and the time × group interaction, with ward and person ID included as a random intercept. Models were adjusted for gender, ethnicity, educational level and BMI due to the significant differences between intervention and control groups observed at baseline.

Secondary continuous outcomes (acceleration, LIPA, sedentary time, cardiometabolic measures, sleep quality, QoL, and PA intention) were analysed using the same mixed-effects framework. Adjusted mean differences (intervention minus control) are reported with 95% confidence intervals (CIs) and p-values. Pre-specified subgroup analyses for gender, age category, education level, occupation, socioeconomic status, BMI category, WHtR, and blood pressure status were conducted by adding a three-way interaction term (time × group × subgroup) to the model. These subgroups were defined a priori based on their established relevance to PA behaviour and health risk [41]. Subgroup-specific effects and p-values for interaction are reported. A two-sided p-value of less than 0.05 was considered statistically significant.

## Results

### Baseline characteristics of participants

Of the 264 participants recruited across 14 clusters, 220 completed follow-up assessments, representing a 16.7% attrition rate. Of the 132 participants per arm, follow-up assessments were completed by 109 participants in the intervention group and 111 in the control group, corresponding to follow-up rates of 82.6% and 84.1%, respectively. Loss of follow-up occurred due to death (2), migration (3), inability to locate participants (5), discontinued participating in the intervention (5), or refusal for follow-up assessment (29). Four participants in each arm had insufficient accelerometer wear time, leaving 212 individuals (105 intervention, 107 control) having sufficient accelerometer data to derive PA outcomes. (Fig. 1). Of those allocated to the intervention arm, five discontinued participation during the intervention period. All remaining participants attended all three scheduled home visits, and FCHVs delivered the intended session content as planned.

The mean (SE) age was 48.8 (1.2) years in the control group and 50.5 (1.0) years in the intervention group. Women comprised 73.5% of the control group and 61.4% of the intervention group. Most participants were married (93.2% in the control group and 97% in the intervention group), and approximately two-thirds had completed their secondary education. Body mass index averaged 26.0 kg/m^2^ (SE 0.4) in the control group and 27.2 kg/m^2^ (SE 0.4) in the intervention group. Systolic and diastolic blood pressures were comparable between groups. Baseline PA levels were also similar, with mean (SE) non-bouted MVPA at 131.7 (4.8) min/day in control and 133.3 (5.3) min/day in intervention groups (Table 1). At baseline, the intervention group included a higher proportion of men, higher mean BMI, and lower sleep quality than control group. The control group had a higher proportion of participants with higher education. No other baseline characteristics differed significantly between the groups.

**Table 1:** Baseline demographic, clinical, and physical activity characteristics of participants in the control and intervention groups (intention-to-treat population)

Characteristics		Participants, No (%)		
		Control group(*n* = 132)	Intervention group (*n* = 132)	*p*-value
Age category	up to 30	10 (7.6)	9 (6.8)	0.427
	30—44	43 (32.6)	34 (25.8)	
	45 and above	79 (59.8)	89 (67.4)	
	Age, mean (SE), Years	48.8 (1.2)	50.5 (1.0)	0.280
Gender	Male	35 (26.5)	51 (38.6)	0.036
	Female	97 (73.5)	81 (61.4)	
Marital status	Married	123 (93.2)	128 (97)	0.255
	Unmarried	9 (6.8)	4 (3.0)	
Education level	Illiterate	30 (22.7)	34 (25.8)	0.043
	School Education	86 (65.2)	93 (70.5)	
	Bachelor or above	16 (12.1)	5 (3.8)	
Ethnicity	Brahmin/Chhetri	97 (73.5)	63 (47.7)	< 0.001
	Dalit	11 (8.3)	28 (21.2)	
	Janajati	24 (18.2)	41 (31.1)	
Occupation	Agriculture	37 (28.0)	29 (22.0)	0.124
	Employee	27 (20.5)	28 (21.2)	
	Housewife or husband	53 (40.2)	68 (51.5)	
	Others	15 (11.4)	7 (5.3)	
Family type	Nuclear	81 (61.4)	82 (62.1)	0.899
	Joint/Extended	51 (38.6)	50 (37.9)	
Socioeconomic class	Lower/Upper Lower	38 (28.8)	40 (30.3)	0.964
	Lower Middle	56 (42.4)	55 (41.7)	
	Upper/Upper Middle	38 (28.8)	37 (28.0)	
Clinical Characteristics, mean (SE)^†^	Weight, kg	62.7 (1)	66.2 (1)	0.016
	BMI, kg/m^2^	26 (0.4)	27.2 (0.4)	0.032
	WHtR	0.6 (0)	0.6 (0)	0.068
	SBP, mm Hg	124.5 (1.9)	124.8 (1.8)	0.888
	DBP, mm Hg	83.4 (1.1)	84 (1)	0.695
Physical activity, mean (SE)^‡^	Acceleration, mg	34.2 (0.8)	34.8 (0.9)	0.586
	MVPA 10 min bout, min/day	22.1 (2.1)	21.1 (1.9)	0.718
	MVPA non-bout, min/day	131.7 (4.8)	133.3 (5.3)	0.824
	LIPA, min/day	149.9 (3.8)	152 (4.5)	0.718
	Sedentary, hours/day	5.2 (0.2)	5.1 (0.3)	0.780
Intention, mean (SE)^†^	Intention	4 (0.1)	4.2 (0.1)	0.125
Sleep^‡^	Quality > = 80%	103 (81.7%)	94 (72.9%)	0.091
	Quality < 80%	23 (18.3%)	35 (27.1%)	
Sleep, mean (SE)^‡^	Duration, hours/day	5.4 (0.1)	5.3 (0.1)	0.833
Quality of life, mean (SE) ^†^	Physical health	15.7 (0.2)	15.6 (0.2)	0.727
	Psychological health	14.9 (0.2)	14.7 (0.1)	0.293
	Social relations	15 (0.1)	14.8 (0.1)	0.186
	Environment	14.9 (0.1)	14.9 (0.1)	0.710

*SE* standard error, *MVPA* moderate to vigorous physical activity, *LIPA* light-intensity physical activity, *SBP* systolic blood pressure, *DBP* diastolic blood pressure, *BMI* body mass index, *WHtR* waist to height ratio, *QoL* quality of life

^†^Multiple imputation was performed for 43 participants

^‡^Multiple imputation was performed for 51 participants

### Effect of the intervention on primary outcome

#### MVPA minutes per day

**Fig. 2:**
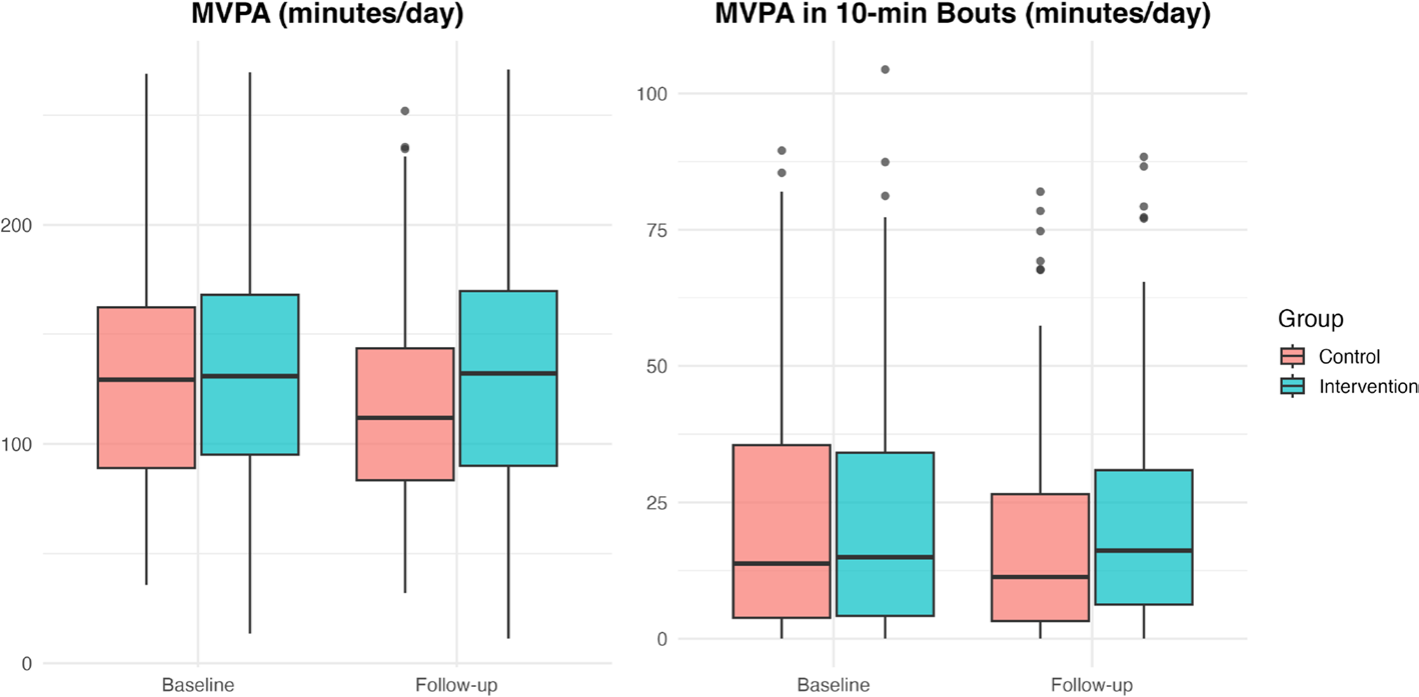
MVPA minutes (non-bout and 10 min bout) in both groups during two different time points

In the intention-to-treat principle analysis, including the loss to follow-up participants, both groups experienced a decline in daily MVPA over the study period. The decrease in non-bout MVPA was greater in the control group, with a mean (SE) of 14.1 (3.1) minutes per day compared with 4.3 (3.7) minutes per day in the intervention group, showing a more modest decline in non-bout MVPA in the intervention arm compared with controls (Fig. 2). The adjusted between-group difference in change in non-bout MVPA was 9.8 min (95% CI 0.41 to 19.18, *p* = 0.041). Similar trends were observed for MVPA accumulated in bouts of at least 10 min with an adjusted difference of 4.53 min/day (95% CI 0.29 to 8.77, *p* = 0.036) (Table 2).

**Table 2:** Mean (SE) primary and secondary outcome (intention-to-treat)

Variable	Timepoint	Control	Intervention	Estimate (95% CI)	*p*-value
Primary Outcomes					
MVPA min/day non bout^‡^	Baseline	131.7 (4.8)	133.3 (5.3)		
	Follow-up	118.1 (4.2)	131.3 (5.1)		
	6-month, Change	−14.1 (3.1)	−4.3 (3.7)	9.8 (0.41, 19.18)	0.041
MVPA min/day 10 min bout^‡^	Baseline	22.1 (2.1)	21.1 (1.9)		
	Follow-up	17.4 (1.7)	21.3 (1.8)		
	6-month, Change	−5.1 (1.6)	−0.6 (1.4)	4.53 (0.29, 8.77)	0.036
Secondary Outcomes					
Acceleration, mg^‡^	Baseline	34.2 (0.8)	34.8 (0.9)		
	Follow-up	31.5 (0.7)	34.3 (0.8)		
	6-month, Change	−2.7 (0.6)	−0.9 (0.7)	1.84 (0.13, 3.55)	0.035
LIPA, min/day^‡^	Baseline	149.9 (3.8)	152 (4.5)		
	Follow-up	142.2 (3.8)	152.3 (4.4)		
	6-month, Change	−7.8 (2.7)	−0.7 (3.1)	7.14 (−1.03, 15.3)	0.086
Sedentary, min/day^‡^	Baseline	313.9 (12.9)	308 (16.6)		
	Follow-up	345.4 (13.4)	307.3 (15.3)		
	6-month, Change	32.5 (10.9)	3.1 (11.6)	−29.45 (−60.69, 1.78)	0.064
Sleep quality^‡^	Baseline	84.4 (0.6)	82.4 (0.7)		
	Follow-up	84 (0.7)	83.5 (0.6)		
	6-month, Change	−0.5 (0.6)	1.1 (0.5)	1.64 (0.09, 3.2)	0.038
Sleep duration, min/day^‡^	Baseline	321.1 (5.2)	319.4 (6)		
	Follow-up	326.8 (5.5)	326.8 (5.5)		
	6-month, Change	5.3 (4.3)	7.5 (4.2)	2.2 (−9.64, 14.03)	0.715
SBP, mm Hg^†^	Baseline	124.5 (1.9)	124.8 (1.8)		
	Follow-up	122.1 (1.7)	124.1 (1.6)		
	6-month, Change	−1.9 (1.3)	−0.8 (1.1)	1.04 (−2.33, 4.41)	0.543
DBP, mm Hg^†^	Baseline	83.4 (1.1)	84 (1)		
	Follow-up	81.7 (1)	82.3 (1)		
	6-month, Change	−1.4 (0.9)	−1.8 (0.7)	−0.34 (−2.57, 1.89)	0.765
Weight, Kg^†^	Baseline	62.7 (1)	66.2 (1)		
	Follow-up	62.5 (1)	66.4 (1)		
	6-month, Change	−0.3 (0.3)	0 (0.3)	0.28 (−0.48, 1.03)	0.474
BMI^†^	Baseline	26 (0.4)	27.2 (0.4)		
	Follow-up	25.8 (0.4)	27.1 (0.4)		
	6-month, Change	−0.2 (0.1)	−0.2 (0.1)	0.03 (−0.35, 0.4)	0.890
WHtR^†^	Baseline	0.6 (0)	0.6 (0)		
	Follow-up	0.6 (0)	0.6 (0)		
	6-month, Change	0 (0)	0 (0)	0 (−0.01, 0.01)	0.755
Intention^†^	Baseline	4 (0.1)	4.2 (0.1)		
	Follow-up	3.8 (0.1)	4 (0.1)		
	6-month, Change	−0.2 (0.1)	−0.2 (0.1)	0.06 (−0.14, 0.26)	0.549
Qol environment^†^	Baseline	14.9 (0.1)	14.9 (0.1)		
	Follow-up	14.4 (0.1)	14.7 (0.1)		
	6-month, Change	−0.4 (0.2)	−0.2 (0.2)	0.2 (−0.23, 0.63)	0.349
Qol physical^†^	Baseline	15.7 (0.2)	15.6 (0.2)		
	Follow-up	15.4 (0.2)	15.6 (0.2)		
	6-month, Change	−0.3 (0.2)	0 (0.2)	0.33 (−0.17, 0.84)	0.196
Qol psychological^†^	Baseline	14.9 (0.2)	14.7 (0.1)		
	Follow-up	14.5 (0.1)	14.7 (0.1)		
	6-month, Change	−0.5 (0.2)	0 (0.1)	0.46 (0.01, 0.9)	0.046
Qol social^†^	Baseline	15 (0.1)	14.8 (0.1)		
	Follow-up	14.4 (0.1)	14.6 (0.2)		
	6-month, Change	−0.7 (0.2)	−0.2 (0.2)	0.48 (0.01, 0.95)	0.045

*SE* standard error, *MVPA* moderate to vigorous physical activity, *LIPA* light-intensity physical activity, *SPB* systolic blood pressure, *DBP* diastolic blood pressure, *BMI* body mass index, *WHtR* waist to height ratio, *QoL* quality of life

^†^Multiple imputation was performed for 43 participants

^‡^Multiple imputation was performed for 51 participants

The results were similar in the Per protocol analysis, showing a 13.40 min/day difference (95% CI 2.30 to 24.51, *p* = 0.018) for non-bout MVPA, and a difference of 6.1 min/day (95% CI 0.87 to 11.34, *p* = 0.023) for MVPA in 10-min bout, favouring the intervention. (Supplementary Table 2).

#### Other physical activity metrics

Acceleration values fell slightly in both groups, with a between-group difference of 1.84 mg (95% CI 0.13 to 3.55, *p* = 0.035). No statistically significant between-group differences were found for LIPA, although the intervention group had a smaller reduction (− 0.7 vs − 7.8 min/day, *p* = 0.086). Sedentary time increased by 32.5 min/day in controls and only 3.1 min/day in the intervention group, with a difference of − 29.45 min/day (*p* = 0.064) (Table 2).

#### Subgroup analyses of intervention effects on MVPA (non-bout)

**Fig. 3:**
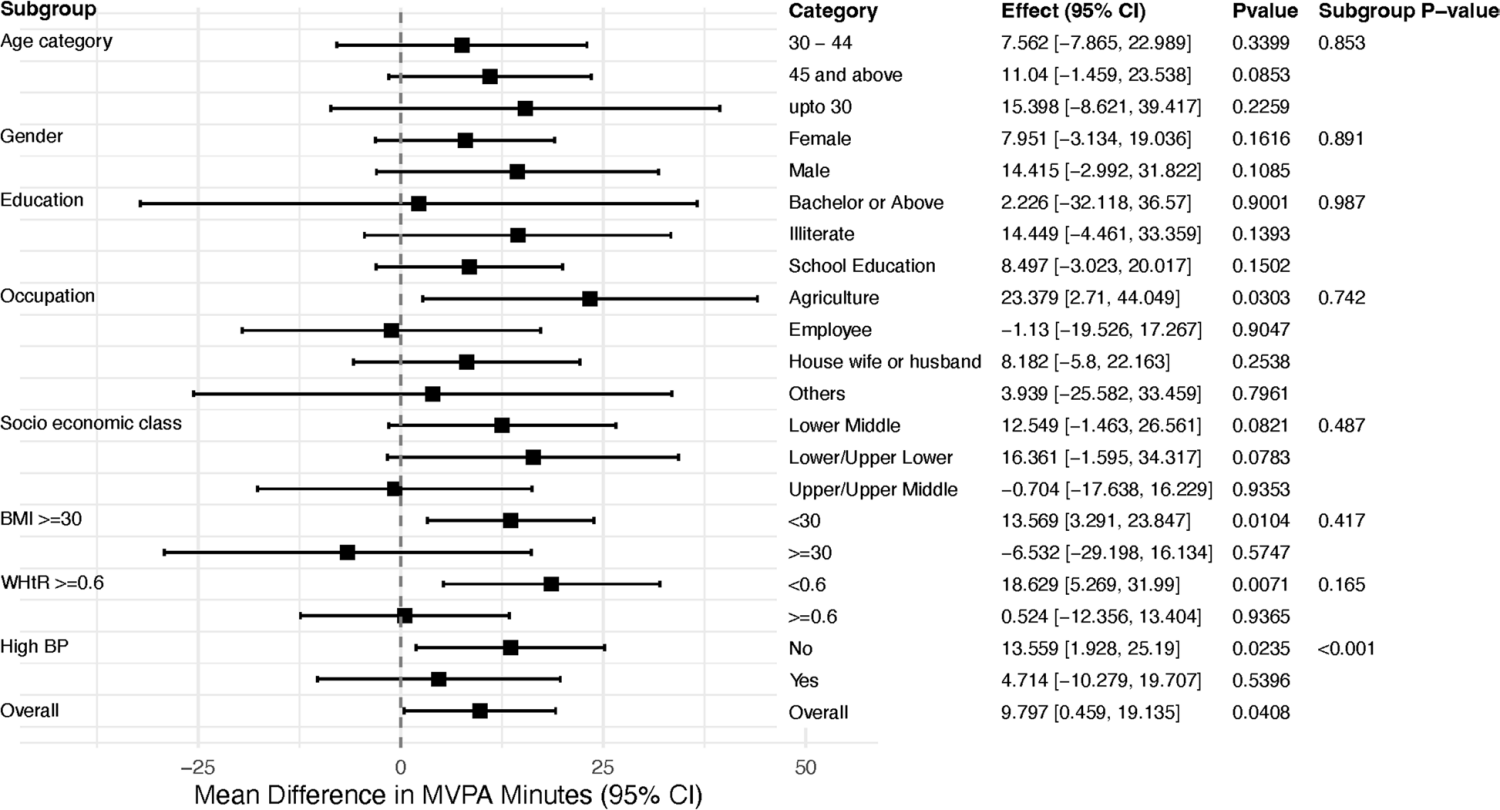
Forest plot of the difference of mean MVPA min (non-bout) by subgroup

The subgroup analyses were conducted for gender, age category, education level, occupation, socioeconomic status, BMI category, WHtR, and blood pressure status. No subgroup exhibited a statistically significant adjusted between-group difference in non-bout MVPA except for high BP (*p* < 0.001) (Fig. 3).

### Effect of intervention on secondary outcomes

Sleep quality improved slightly in the intervention arm (+ 1.1%) but declined marginally in controls (− 0.5%), producing an adjusted between-group difference of 1.64% (95% CI 0.09 to 3.20, *p* = 0.038). Sleep duration increased by 7.5 min/day in the intervention group versus 5.3 min/day in controls, but the difference was not significant (*p* = 0.715). Changes in systolic and diastolic blood pressure were small in both groups (SBP: − 0.8 vs − 1.9 mmHg, DBP: − 1.8 vs − 1.4 mmHg) with no significant differences. Weight and BMI remained stable across arms, with mean changes close to zero. WHtR was unchanged in both groups (0.60 baseline and follow-up). Intention scores decreased slightly in both groups (− 0.2 points each, *p* = 0.549). Between-group difference in the environment and physical health domain of Quality-of-life measures was non-significant, while psychological and social domains showed a significant difference (*p* < 0.05) (Table 2).

## Discussion

This cluster randomised controlled trial was designed to test whether a three-month, FCHV-led, home-based educational intervention could increase device-measured MVPA over six months among adults in semi-urban Nepal. This a priori hypothesis was not supported, as both arms experienced reductions in MVPA. However, in the intention-to-treat analysis, the decline was substantially smaller in the intervention group. Compared with the control group, the intervention group experienced a 9.8 min/day smaller decline in non-bout MVPA and a 4.53 min/day smaller decline in 10-min bout MVPA, indicating that intervention participants maintained higher PA levels at follow-up than those receiving usual care.

Although MVPA declined in both arms from baseline to follow-up, the reduction was smaller in the intervention group. By the end of the trial, participants in the intervention arm maintained more of their baseline PA, while the control group experienced a steeper drop. In relative terms, the intervention group retained about 70% more non-bout MVPA and nearly 90% more bouted MVPA compared with controls. Such downward trajectories are consistent with device-based cohorts such as CARDIA and European follow-up studies, where MVPA typically decreases and sedentary time increases over time [42–44]. This pattern also aligns with broader evidence from LMICs, where PA promotion interventions rarely increase total MVPA and instead tend to yield modest effects or slow the decline over time [45]. At baseline, participants may have overperformed because of the novelty of accelerometer wear or the Hawthorne effect, while at follow-up, they likely returned to more typical behaviour [44, 45]. Because both groups were exposed to the same influences, the smaller decline in the intervention arm reflects a genuine programme effect. Preserving about ten extra minutes of daily MVPA is clinically meaningful, as large pooled analyses show even modest device-measured increases are linked to lower cardiovascular and mortality risk [4]. 

### Comparison with other community-based interventions

Few trials in LMICs have used device-measured PA comparable to ours. SMART-STEP cluster RCT in India targeted desk-based employees by combining smartphone prompts and pedometer monitoring, which resulted in increased device-measured PA [46]. Although delivered in a workplace setting, the intervention, like ours, relied on simple behavioural prompts embedded in existing systems, illustrating the value of low-cost, scalable strategies. Similarly, a trial among Pakistani-origin men tested a culturally adapted community programme and found device-measured increases in PA after six months [47]. While not conducted in an LMIC, the study highlights that community-delivered, culturally tailored approaches can help maintain PA levels in populations at risk of inactivity.

Within Nepal, FCHV-led community-based health promotion (COBIN trials) with PA as a secondary outcome and having PA educational component as part of the intervention has demonstrated to improve cardiovascular risk factors, including reductions in blood pressure and improved glycaemic control in adults with type 2 diabetes [20, 21]. However, these studies relied primarily on self-reported PA outcomes and did not show significant PA change. Our trial adds to this literature by using accelerometry to quantify PA changes objectively, reducing recall bias and increasing measurement precision [48].

Subgroup analyses for BMI and WHtR did not reach conventional statistical significance but showed a consistent pattern of larger intervention effects among participants with lower BMI and WHtR. The interaction p-value for WHtR (*p* = 0.165), while not statistically significant, could be viewed as borderline in the context of exploratory subgroup analyses, which are often underpowered and conservative. These findings should be interpreted cautiously and not as confirmatory. However, the trend is informative: individuals with higher adiposity, who might be expected to benefit most from increased PA, appeared to experience smaller intervention effects [49]. This may indicate greater physical, motivational, or environmental barriers to behaviour change in these groups [41]. Future interventions may require more intensive tailoring or structural support to better engage individuals with higher BMI or central adiposity.

### Implications for practice and policy

The findings have important implications for public health practice, particularly in LMIC contexts where access to structured exercise programmes is limited. Globally, WHO’s Global Action Plan on PA highlights community-based, culturally adapted approaches as cost-effective strategies for reducing inactivity [15]. This trial reinforces that established cadres like FCHVs can feasibly deliver targeted PA promotion with measurable benefits in semi-urban Nepal.

The scalability of this model is a major strength. FCHVs are embedded within Nepal’s primary health care system, providing nationwide coverage and strong community trust. Integrating PA promotion into existing FCHV responsibilities, alongside maternal, child health, and NCD prevention activities, offers an efficient, sustainable solution. However, this would require added support, clear task prioritization, and possibly incentives to avoid overburdening an already stretched workforce [50]. The success of FCHV-led programs like NCD management suggests that, with adequate training and support, their roles can effectively expand [20–22]. Integrating PA counselling could be efficient and sustainable, but only with policy measures addressing workload, recognition, and remuneration. In resource-limited settings, this model could be a pragmatic alternative to infrastructure-intensive interventions like gyms or sports facilities, which often fail to reach disadvantaged populations. From a policy perspective, embedding such programmes within national NCD action plans and aligning them with global PA targets could accelerate progress toward commitments like Nepal’s goal of a relative reduction in insufficient PA by 15% by 2030 [16]. Demonstrating cost-effectiveness, equity, and sustainability will be essential for securing policy support and resources for scale-up.

### Strengths and limitations

This trial has several notable strengths. The intervention was co-designed with community members and guided by behavioural theory, ensuring cultural relevance and practicality in the semi-urban Nepalese context. The engagement of FCHVs, an established and trusted cadre within the primary health care system, enhanced feasibility, acceptability, and scalability. PA was measured objectively using wrist-worn accelerometers, minimising recall and social desirability bias common in self-reported measures. The cluster randomised design, high follow-up rate (83.4%), and application of intention-to-treat analysis with multiple imputation further strengthened internal validity. Integration of the intervention into an existing health system framework also demonstrates its potential for real-world implementation without requiring major structural changes.

This study has some limitations that must be acknowledged. The relatively short follow-up period of six months limits understanding of the sustainability of PA changes and their potential downstream health effects. While the sample size was adequate for detecting the primary outcome, it may have been insufficient to identify smaller changes in secondary outcomes such as cardiometabolic risk factors and quality of life. Data collection at baseline and follow-up occured in comparable seasons, and thus seasonal variation was unlikely to have materially influenced participants' physical activity levels. Participants, intervention providers and data analysts were not masked, introducing potential bias in subjective outcomes such as sleep quality and quality of life. Intervention providers were aware of allocation, which could have influenced counselling intensity. However, our primary outcomes were device-based and therefore less vulnerable to such bias. Lack of blinding is common in behavioural trials, including LMICs settings where community health workers deliver interventions [46, 51, 52]. Formal participant satisfaction measures were not collected as part of this effectiveness trial. However, acceptability and participant feedback were explored qualitatively during the implementation phases and are reported in a separate paper [32].

### Future directions

Future research should examine strategies for sustaining PA gains over longer periods, adapt interventions for subgroups with smaller effects, and evaluate cost-effectiveness for national or regional scale-up. Incorporating digital tools, peer-support mechanisms, or community group activities alongside FCHV-led home visits may enhance engagement and long-term adherence. These interventions should be driven by community members and facilitated by local government (municipalities). Longitudinal studies linking behavioural changes to cardiometabolic outcomes would also strengthen the case for policy adoption and resource allocation.

## Conclusions

This FCHV-led, community-based educational intervention did not lead to an increase in MVPA but significantly attenuated the declines observed over six months compared with usual care. These findings indicate that community-based education delivered through existing health volunteer platforms may play a role in supporting the maintenance of PA and related movement behaviours in semi-urban populations, particularly in settings undergoing rapid social and lifestyle transitions. Such approaches may complement broader public health strategies aimed at preventing physical inactivity in LMICs.

## Supplementary Information

Supplementary Material 1.

Supplementary Material 2.

Supplementary Material 3.

Supplementary Material 4.

Supplementary Material 5.

## Data Availability

Data relevant to the study are included in the article and uploaded as supplementary files.

## Abbreviations

BMI: Body mass index
BP: Blood pressure
COBIN: Community-Based Intervention for Management of Non-Communicable Diseases
DBP: Diastolic blood pressure
ENMO: Euclidean Norm Minus One
FCHVs: Female Community Health Volunteers
LIPA: Light-intensity physical activity
LMICs: Low- and middle-income countries
MVPA: Moderate-to-vigorous physical activity
NCDs: Non-communicable diseases
PA: Physical activity
SBP: Systolic blood pressure
SES: Socioeconomic status
WHO: World Health Organization
WHOQOL-BREF: World Health Organization Quality of Life—BREF
WHtR: Waist-to-height ratio
