# Supplementary material for: Effectiveness of a female community health volunteer-led physical activity education intervention on accelerometer-derived outcomes in semi-urban Nepal: an open-label, cluster randomised controlled trial
Source: Int J Behav Nutr Phys Act. 2026 Feb 23;23:34. doi: 10.1186/s12966-026-01894-0 (PMC13049831; doi:10.1186/s12966-026-01894-0)
Supplement: Supplementary file 2 — Supplementary Material 2. [file 12966_2026_1894_MOESM2_ESM.docx]

**Supplementary Table 1. Description of the FCHV-led Physical Activity Intervention**

| **Items** | **Description** |
| --- | --- |
| **Brief name** | Female Community Health Volunteer (FCHV)-led community-based physical activity education intervention |
| **Why (rationale, theory)** | The intervention was designed to promote physical activity by addressing behavioural determinants guided by the Theory of Planned Behaviour (TPB), targeting attitudes, subjective norms, and perceived behavioural control to strengthen intention and support integration of physical activity into daily life. |
| **What (materials)** | Intervention checklist, facilitator manual, flipcharts, participant brochures, and demonstration guides for simple home-based physical activities. |
| **What (procedures)** | Family-based, face-to-face home visits delivered by trained FCHVs. Sessions included interactive discussions, demonstrations, and practical guidance on integrating physical activity into daily routines. |
| **Who provided** | Female Community Health Volunteers (n=14) trained through a three-day interactive programme delivered by the principal investigator, a general physician, and a public health professional experienced in community-based NCD management. |
| **How** | In-person household visits involving participants and available family members; reinforcement through monthly SMS or phone calls during the intervention period. |
| **Where** | Participants’ households in semi-urban wards of Pokhara Metropolitan City, Nepal. |
| **When and how much** | Three home visits conducted once per month over three months; each session lasted approximately 1.5 hours. No intervention contact occurred between month 3 and the 6-month follow-up. |
| **Tailoring** | Sessions were delivered at the household level and adapted to participants’ identified barriers, available resources, and daily routines, with encouragement to form family or community walking groups. |
| **Modifications** | No modifications to the intervention content or delivery occurred during the trial. |
| **How well (planned fidelity)** | FCHVs followed a structured intervention checklist. |
| **How well (actual fidelity)** | Detailed assessment of training outcomes, fidelity, and participant feedback is reported in a separate companion paper (under review). |
