# Supplementary material for: Effectiveness of a female community health volunteer-led physical activity education intervention on accelerometer-derived outcomes in semi-urban Nepal: an open-label, cluster randomised controlled trial
Source: Int J Behav Nutr Phys Act. 2026 Feb 23;23:34. doi: 10.1186/s12966-026-01894-0 (PMC13049831; doi:10.1186/s12966-026-01894-0)
Supplement: Supplementary file 3 — Supplementary Material 3. [file 12966_2026_1894_MOESM3_ESM.docx]

**Supplemental Table 2. Mean (SE) primary and secondary outcome per protocol principle**

| **Variable** | **Timepoint** | **Control** | **Intervention** | **Estimate (95% CI)** | **p-value** |
| --- | --- | --- | --- | --- | --- |
| **Primary Outcome** | | | | | |
| MVPA non bout | Baseline | 130.2 (5) | 131.6 (5.7) |  |  |
|  | Follow-up | 114 (4.1) | 127.7 (5.4) |  |  |
|  | Change, 6 months | -16.2 (3.5) | -4 (4.4) | 13.4 (2.3-24.51) | 0.018 |
| MVPA 10 min bout | Baseline | 20.7 (2.2) | 20.8 (2.1) |  |  |
|  | Follow-up | 14.5 (1.5) | 20.5 (1.9) |  |  |
|  | Change, 6 months | -6.1 (2) | -0.2 (1.7) | 6.1 (0.87-11.34) | 0.023 |
| **Secondary Outcome** | | | | | |
| ENMO | Baseline | 34 (0.9) | 34.4 (1) |  |  |
|  | Follow-up | 30.9 (0.7) | 33.6 (0.9) |  |  |
|  | Change, 6 months | -3.2 (0.7) | -0.8 (0.8) | 2.51 (0.52-4.5) | 0.014 |
| LIPA | Baseline | 153.1 (4.2) | 150.5 (5.2) |  |  |
|  | Follow-up | 144.6 (4.4) | 149.4 (5) |  |  |
|  | Change, 6 months | -8.5 (3.2) | -1.1 (3.9) | 8.15 (-1.74-18.05) | 0.106 |
| Sedentary | Baseline | 309.8 (14.3) | 311.7 (17.9) |  |  |
|  | Follow-up | 345.3 (15) | 314.6 (16.1) |  |  |
|  | Change, 6 months | 35.5 (12.6) | 2.9 (14.6) | -37.53 (-75.55-0.5) | 0.053 |
| Sleep quality | Baseline | 84.4 (0.7) | 82.1 (0.9) |  |  |
|  | Follow-up | 83.9 (0.8) | 83.6 (0.6) |  |  |
|  | Change, 6 months | -0.5 (0.8) | 1.5 (0.6) | 2.1 (0.19-4.01) | 0.032 |
| Sleep duration | Baseline | 317.9 (5.9) | 318.8 (6.5) |  |  |
|  | Follow-up | 325 (6.5) | 328.3 (5.9) |  |  |
|  | Change, 6 months | 7.1 (5.4) | 9.5 (5.3) | 3.87 (-10.63-18.37) | 0.600 |
| DBP | Baseline | 126.7 (2.2) | 125.8 (2.1) |  |  |
|  | Follow-up | 124 (2.1) | 124.8 (2) |  |  |
|  | Change, 6 months | -2.6 (1.6) | -1 (1.4) | 1.42 (-2.65-5.49) | 0.492 |
| SBP | Baseline | 84.4 (1.3) | 84.1 (1.2) |  |  |
|  | Follow-up | 82.3 (1.2) | 81.9 (1.1) |  |  |
|  | Change, 6 months | -2.1 (1.1) | -2.2 (0.9) | -0.21 (-2.89-2.47) | 0.876 |
| Weight | Baseline | 63 (1.1) | 67.2 (1.2) |  |  |
|  | Follow-up | 62.8 (1.1) | 67.2 (1.2) |  |  |
|  | Change, 6 months | -0.2 (0.3) | 0 (0.3) | 0.27 (-0.36-0.91) | 0.398 |
| BMI | Baseline | 26.3 (0.4) | 27.5 (0.4) |  |  |
|  | Follow-up | 26.1 (0.4) | 27.3 (0.4) |  |  |
|  | Change, 6 months | -0.2 (0.1) | -0.2 (0.2) | -0.01 (-0.47-0.45) | 0.971 |
| Waist Height Ratio | Baseline | 0.6 (0) | 0.6 (0) |  |  |
|  | Follow-up | 0.6 (0) | 0.6 (0) |  |  |
|  | Change, 6 months | 0 (0) | 0 (0) | 0 (-0.01-0.01) | 0.822 |
| Intention | Baseline | 4 (0.1) | 4.2 (0.1) |  |  |
|  | Follow-up | 3.8 (0.1) | 4 (0.1) |  |  |
|  | Change, 6 months | -0.3 (0.1) | -0.2 (0.1) | 0.06 (-0.18-0.3) | 0.597 |
| QolLenvironment | Baseline | 14.8 (0.2) | 14.9 (0.1) |  |  |
|  | Follow-up | 14.3 (0.2) | 14.6 (0.2) |  |  |
|  | Change, 6 months | -0.6 (0.2) | -0.3 (0.2) | 0.29 (-0.24-0.81) | 0.283 |
| Qol physical | Baseline | 15.6 (0.2) | 15.6 (0.2) |  |  |
|  | Follow-up | 15.3 (0.2) | 15.6 (0.2) |  |  |
|  | Change, 6 months | -0.4 (0.2) | 0 (0.2) | 0.4 (-0.23-1.02) | 0.211 |
| Qol psychological | Baseline | 15 (0.2) | 14.7 (0.2) |  |  |
|  | Follow-up | 14.4 (0.2) | 14.7 (0.2) |  |  |
|  | Change, 6 months | -0.6 (0.2) | -0.1 (0.2) | 0.52 (-0.03-1.07) | 0.063 |
| Qol social | Baseline | 15.1 (0.1) | 14.8 (0.1) |  |  |
|  | Follow-up | 14.2 (0.2) | 14.6 (0.2) |  |  |
|  | Change, 6 months | -0.8 (0.2) | -0.2 (0.2) | 0.59 (0.04-1.15) | 0.037 |
