# Supplementary material for: Effectiveness of a female community health volunteer-led physical activity education intervention on accelerometer-derived outcomes in semi-urban Nepal: an open-label, cluster randomised controlled trial
Source: Int J Behav Nutr Phys Act. 2026 Feb 23;23:34. doi: 10.1186/s12966-026-01894-0 (PMC13049831; doi:10.1186/s12966-026-01894-0)
Supplement: Supplementary file 4 — Supplementary Material 4. [file 12966_2026_1894_MOESM4_ESM.pdf]

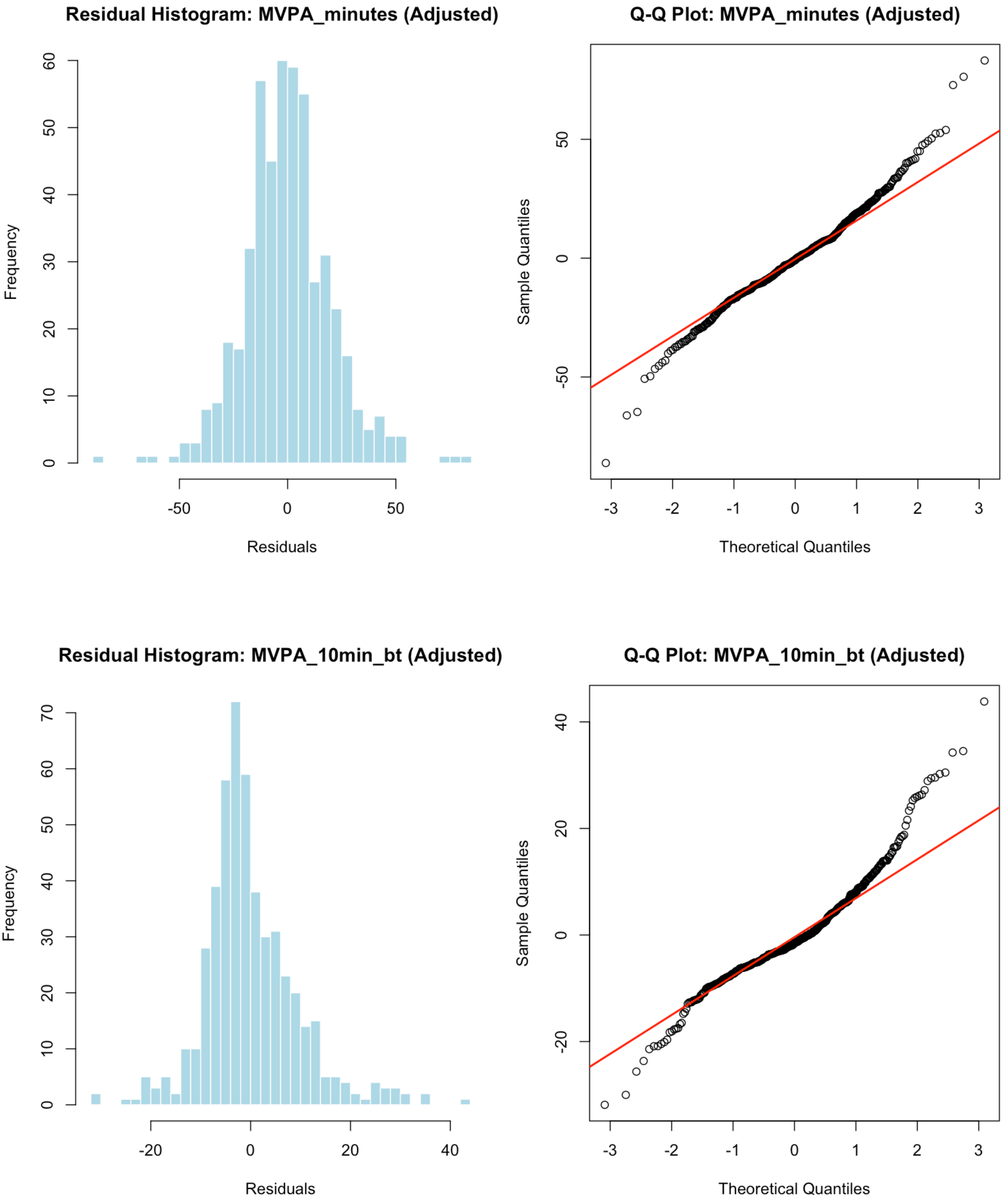

Fig. 1. Residual histogram and Q-Q plot MVPA non-bout and 10 minutes bout
